# Prediction of short-acting beta-agonist usage in patients with asthma using temporal-convolutional neural networks

**DOI:** 10.1093/jamiaopen/ooad091

**Published:** 2023-10-26

**Authors:** Nicholas Hirons, Angier Allen, Noah Matsuyoshi, Jason Su, Leanne Kaye, Meredith A Barrett

**Affiliations:** Propeller Health, San Francisco, CA, United States; ResMed Science Center, San Diego, CA, United States; Propeller Health, San Francisco, CA, United States; School of Public Health, University of California Berkeley, Berkeley, CA, United States; ResMed Science Center, San Diego, CA, United States; ResMed Science Center, San Diego, CA, United States

**Keywords:** asthma, supervised machine learning, telemetry, neural networks, computer

## Abstract

**Objective:**

Changes in short-acting beta-agonist (SABA) use are an important signal of asthma control and risk of asthma exacerbations. Inhaler sensors passively capture SABA use and may provide longitudinal data to identify at-riskpatients. We evaluate the performance of several ML models in predicting daily SABA use for participants with asthma and determine relevant features for predictive accuracy.

**Methods:**

Participants with self-reported asthma enrolled in a digital health platform (Propeller Health, WI), which included a smartphone application and inhaler sensors that collected the date and time of SABA use. Linear regression, random forests, and temporal convolutional networks (TCN) were applied to predict expected SABA puffs/person/day from SABA usage and environmental triggers. The models were compared with a simple baseline model using explained variance (*R*^2^), as well as using average precision (AP) and area under the receiving operator characteristic curve (ROC AUC) for predicting days with ≥1–10 puffs.

**Results:**

Data included 1.2 million days of data from 13 202 participants. A TCN outperformed other models in predicting puff count (*R*^2^ = 0.562) and day-over-day change in puff count (*R*^2^ = 0.344). The TCN predicted days with ≥10 puffs with an ROC AUC score of 0.952 and an AP of 0.762 for predicting a day with ≥1 puffs. SABA use over the preceding 7 days had the highest feature importance, with a smaller but meaningful contribution from air pollutant features.

**Conclusion:**

Predicted SABA use may serve as a valuable forward-looking signal to inform early clinical intervention and self-management. Further validation with known exacerbation events is needed.

## Introduction

Asthma contributes to significant morbidity, mortality, medical cost, and social burden globally.^1^ In the United States, asthma is associated with over $50.1 billion in annual direct medical spend,^2^ not including the 14 million days of work and school missed.^3^ Much of this cost is driven by acute worsening events such as exacerbations; early identification of such worsening could enable proactive intervention and possible prevention.

In asthma treatment, short-acting beta-agonists (SABA), sometimes referred to as rescue medications, are inhaled medications used for acute relief of symptoms such as shortness of breath, wheezing, coughing, or difficulty breathing. SABA use is an important marker of respiratory disease status such as asthma control^4^ and increased SABA use has been associated with worsening symptoms and acute exacerbations.^5^ As such, incremental increases in SABA use from baseline could serve as a real-time indicator of impending exacerbations. In addition, overuse of these medications (>2 puffs per day) can lead to adverse effects.^6^^,^^7^

Traditional SABA data sources have included pharmacy fill records, patient-reported diaries, or non-connected inhaler dose counters, which can require presentation to a clinician for evaluation, time-consuming record-keeping, or time-lagged reports from claims.^8^ Digital tools have emerged to capture SABA use objectively and passively. Passive monitoring of SABA use with inhaler sensors provides the date, time, and location of use, and offers a more real-time and objective component of evaluating asthma status remotely in between visits.^4^

One unique opportunity with these types of data is the ability to merge geolocated health outcome data within a broader social, environmental, and behavioral context to explore non-clinical factors associated with health outcomes.^9^ Beyond just clinical care, approximately 80% of health drivers fall under the social, behavioral, or environmental determinants of health.^10^ For example, a well-established literature has demonstrated significant associations between air pollution exposure and asthma morbidity.^11^

These unique, large digital health datasets demand that methods keep pace with data availability. A growing application of machine learning (ML) and artificial intelligence (AI) approaches have shown promise in asthma^9^ and other diseases.^12^^,^^13^ As more complex models have been developed, including novel deep learning architectures, explainability remains a core issue impacting trust, adoption, safety, and bias in both public health and clinical settings.^14^ Explainability has therefore emerged as an important area of applied ML research,^15^ and recent advances in model interpretability methods allow practitioners to reduce the “black-box” nature of complex models. One method, Shapley additive explanations (SHAP),^16^ has emerged as a popular model-agnostic toolset for interpreting models.

In this study, we aimed to evaluate the performance of several ML models in predicting daily SABA use for participants with asthma. We leveraged a large SABA use dataset collected by inhaler sensors and then merged with contextual data, to predict daily SABA puff counts as well as classify days with high puff counts associated with asthma worsening. For the best-performing model, we examined the importance of input features including previous SABA use, air pollutants and weather over lagged time periods, using multiple methods to enhance interpretability.

## Methods

### Participants

Participants with self-reported asthma (≥4 years of age) enrolled in a digital health platform (Propeller Health, WI), which included a smartphone application and inhaler sensors that collected the date and time of each SABA puff. Participants were included in the analysis if they used the platform for ≥30 days with ≥1 SABA puff from December 2017 to March 2019. Participants were recruited through social media advertisements (eg, Facebook) and downloaded the app on their smartphone. All participants agreed to Propeller’s Terms of Services, which describe the use of aggregated, deidentified data for research purposes. The retrospective analysis was determined to be exempt by the Copernicus Independent Review Board under Protocol PRH1-18-132 and a waiver of consent provided. A waiver of consent was sought, as the retrospective consent process would likely increase the risk of a privacy threat and place undue burden on the patients, as well as possibly introduce bias into the study.

### Digital health platform

**Figure 1.**
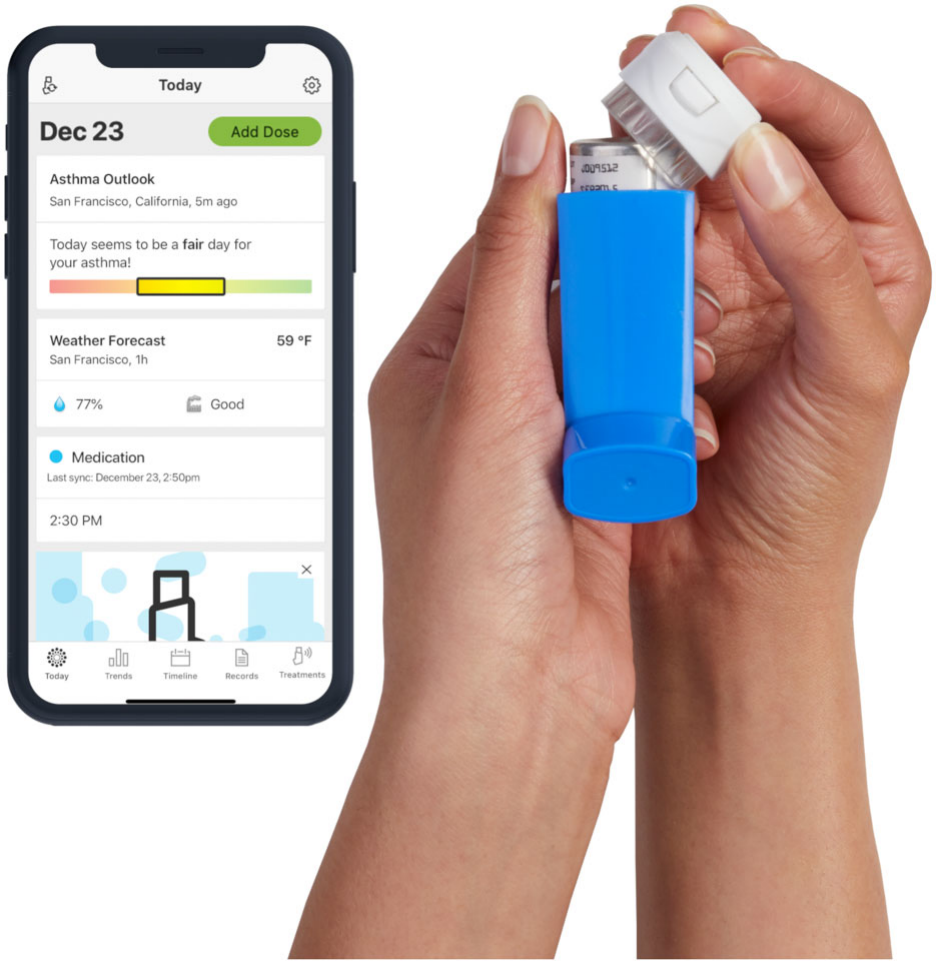
The Bluetooth-enabled sensor attaches to the inhaled medication and can passively monitor the date, time, and location of use. The information is wirelessly transmitted to secure servers, which analyze and provide information back to patients, caregivers, and clinicians.

Propeller Health is a Food and Drug Administration-cleared digital therapeutic comprised of digital sensors that fit onto inhalers, a patient-facing mobile app with reminders and feedback to support self-management, and a clinical provider dashboard to support clinical care (Figure 1). The sensors passively monitor the use of inhaled medications, capturing the date and time of each usage, and approximate geographic location (when paired with a smartphone). These signals can provide an assessment of daily adherence to controller medication therapy and changes in the use of SABA. Clinicians can use the information to inform medication adjustments or early intervention in the care of respiratory diseases including asthma and chronic obstructive pulmonary disease (COPD). The platform has been described in detail previously.^17^^,^^18^

### Data preprocessing

Data were denormalized at a participant-day level and included the count of SABA puffs per participant per day along with their age. Participants needed at least 21 days of data for inclusion, and the first 7 days were dropped to allow the user a learning period for the device. For each user day, the geographic locations for all medication use events as well as sensor syncs were collected via smartphone GPS and assigned environmental information. This process enabled a detailed characterization of participant environmental exposure for each day of participation. Environmental data included weather data (eg, temperature, relative humidity, wind speed) and air pollutants (nitrogen dioxide (NO_2_), ozone (O_3_), sulfur dioxide (SO_2_), and particulate matter of ≤2.5 microns (PM_2.5_) and ≤10 microns (PM_10_)) at hourly intervals. Additional details on the assignment process, input variables, and data processing can be found in the Supplemental Material, including the Environmental assignment information section and Supplementary Table 1.

### Predictive model development

Participants were randomly assigned to a test group (20%) and three cross-validation groups (80%). A linear regression (LR), random forest (RF), and a temporal convolutional network (TCN)^19^ were trained to predict an expected count of SABA puffs/person/day. A TCN is a neural network that contains convolutional layers over the time dimension that may be (1) causal through time, such that an output cannot depend on future timesteps and (2) dilated, such that a filter is applied over a length larger than the input by skipping certain timesteps.^20^ Integration of the time dimension into the model allows for accounting of lags in time from the input features, for example, time lags in the effects of air pollutants.

Candidate models were chosen to reflect increasing model complexity, with LR being the simplest and TCN the most complex. A TCN was selected due to its suitability in predicting sequential data at the individual level,^21^ based on recent physical and environmental factors. First, it allows the high-granularity input of data (at the per variable per day level), rather than requiring aggregation functions over a trailing window that is typically used for other model classes.^22^ Second, the TCN’s causal convolutions ingest and process the data in a way that respects its relative ordering through time, representing how a person is exposed to environmental conditions and pollutants that may accumulate over the short-term. Finally, more complex sequence models such as transformers^23^ did not yield performance improvements.

The model incorporated SABA puff data from the preceding 7 days, along with weather (temperature, precipitation, and wind speed) and air pollutant data (NO_2_, O_3_, SO_2_, PM_2.5_ and PM_10_) from the preceding 7 days and the day of prediction (see Supplementary Table 1). Predictive performance of the final model was evaluated out-of-sample on the test group.

### Model performance evaluation

Performance of the models was evaluated against a baseline model, which assumes that the puff count on the prediction day is equal to the mean of the prior 7 days. Regression performance was evaluated with *R*^2^. Model performance was also assessed with two classification measures related to unsafe SABA dosing: (1) predicting an absolute SABA puff count greater than or equal to thresholds of 1, 2, 4, 6, 8, or 10 daily puffs (with overuse being >2 puffs),^6^^,^^7^ and (2) predicting an increase in SABA puff count from the preceding day to the prediction day greater than or equal to 1, 2, 4, 6, 8, and 10 daily puffs. In both cases, we refer to the set of days where the puff count on the prediction day meets or exceeds the puff threshold as the positive class, and the set of all other days as the negative class. A detailed explanation of the evaluation metrics used can be found in the Supplementary Material.

### Feature interpretation

We used SHAP,^16^ a framework for inferring feature importance from trained models, to visualize the impact of different model inputs over different periods of time. Shapley values are designed to decompose model predictions to their attributable input features after the model is trained. These values were used to examine how input features on the prediction day and preceding 7 days impacted the predicted number of SABA puffs.

## Results

### Participants

Model development included 1.2 million days of data from 13 202 participants. The mean (SD) participant age was 38.3 (16.9) years, mean (SD) SABA puffs/person/day was 1.23 (2.06), 7548 people (57.2%) had a controller medication on plan, and 8823 (89.7%) were considered uncontrolled at baseline per the Asthma Control Test score at enrollment (Table 1). Across United States regions, 35.4%, 25.9%, 21.4%, and 17.3% of the study population were located in the South, Midwest, West, and Northeast, respectively. For the entire study population (*N* = 13 202), the condition-positive rates (CPR) of days with ≥1, 2, 4, 6, 8, and 10 SABA puffs were: 21.9%, 18.5%, 9.5%, 5.2%, 3.1%, and 1.9%, respectively, and the CPR of days with a day-over-day increase of ≥1, 2, 4, 6, 8, and 10 SABA puffs were 13.1%, 9.8%, 3.6%, 1.5%, 0.8%, and 0.4%, respectively. For the subset of participants in the test group (*N* = 2640), the CPR of days with ≥1, 2, 4, 6, 8, and 10 SABA puffs were: 22.6%, 19.1%, 10.0%, 5.5%, 3.5%, and 2.1%, respectively, and the CPR of days with a day-over-day increase of ≥1, 2, 4, 6, 8, and 10 SABA puffs were 13.5%, 10.2%, 3.8%, 1.6%, 0.9%, and 0.5%, respectively.

**Table 1.** Characteristics of the participants included in the TCN model development.

Characteristic	Average/Count	N available	% of total N
Age (mean (SD)); years	38.3 (16.9)	13 202	100.0
Female; *n* (%)	8712 (71.0%)	12 271	92.9
Baseline ACT (mean (SD))	13.9 (4.8)	9836	74.5
Uncontrolled Asthma (ACT < 20); *n* (%)	8823 (89.7%)	9836	74.5
Android; *n* (%)	7548 (57.2%)	13 196	100.0
SABA use (mean (SD)); puffs/day	1.23 (2.06)	13 202	100.0
Participants with a controller medication on plan; *n* (%)	7548 (57.2%)	13 196	100.0
Duration of participation (mean (SD)); days	98.4 (100.8)	13 202	100.0
Region			
South; *n* (%)	4636 (35.4%)	13 087	99.1
Midwest; *n* (%)	3385 (25.9%)	13 087	99.1
West; *n* (%)	2804 (21.4%)	13 087	99.1
Northeast; *n* (%)	2262 (17.3%)	13 087	99.1

Sample sizes varied by characteristic; the full sample included 13 202 participants.

### Model performance

Examining model performance across a range of metrics, the TCN consistently outperformed alternative models (Table 2). In terms of overall variance explained (*R*^2^), the TCN outperformed the baseline model, LR, and RF by 15.7%, 4.4%, and 5.1%, respectively, on a relative basis for predicting SABA puff count (*R*^2^ = 0.562) (Table 2). The TCN also outperformed other models by 45.7%, 11.7%, and 14.8%, respectively, in predicting the day-over-day change in SABA puff count (*R*^2^ = 0.344) (Table 3).

**Table 2.** Predictive performance comparison on the test group in predicting puff count for the baseline model, linear regression (LR), random forest, (RF), and TCN.

	≥Puff threshold	CPR (%)	Baseline	LR	RF	TCN
SABA puffs						
*R* ^2^	–	–	0.490	0.538	0.535	0.562
AP	1	22.6	0.692	0.731	0.726	0.762
	2	19.1	0.679	0.716	0.711	0.747
	4	10.0	0.646	0.681	0.676	0.705
	6	5.5	0.593	0.630	0.626	0.647
	8	3.5	0.531	0.571	0.566	0.585
	10	2.1	0.457	0.497	0.493	0.506
ROC AUC	1	22.6	0.862	0.877	0.872	0.890
	2	19.1	0.871	0.886	0.879	0.897
	4	10.0	0.910	0.923	0.913	0.929
	6	5.5	0.929	0.940	0.932	0.944
	8	3.5	0.936	0.947	0.938	0.950
	10	2.1	0.939	0.950	0.941	0.952

**Table 3.** Predictive performance comparison on the test group in predicting change in puff count of the baseline model, linear regression (LR), random forest (RF), and TCN.

	≥ Puff threshold	CPR (%)	Baseline	LR	RF	TCN
Day over day change in SABA puffs						
*R* ^2^	–	–	0.236	0.308	0.300	0.344
AP	1	13.5	0.308	0.315	0.303	0.369
	2	10.2	0.267	0.278	0.265	0.330
	4	3.8	0.166	0.173	0.156	0.215
	6	1.6	0.101	0.101	0.087	0.124
	8	0.9	0.069	0.073	0.058	0.092
	10	0.5	0.043	0.047	0.034	0.056
ROC AUC	1	13.5	0.681	0.668	0.646	0.697
	2	10.2	0.694	0.685	0.660	0.712
	4	3.8	0.703	0.693	0.656	0.717
	6	1.6	0.687	0.674	0.623	0.698
	8	0.9	0.689	0.684	0.632	0.707
	10	0.5	0.673	0.674	0.621	0.694

The TCN achieved an AP for predicting a symptom day (a day with ≥1 SABA puffs) of 0.762, a noticeable improvement compared to the baseline (0.692) as well as LR (0.731) and RF (0.726) models. Similar improvements were observed when examining higher puff thresholds per day ranging from ≥2 to ≥10. A confusion matrix is provided in Table 4 for classifying puff thresholds of ≥1 and ≥10. When examining a change in SABA puffs/person/day, the TCN predicted any increase in SABA use with an AP of 0.369, an improvement over the baseline (0.308), LR (0.315), and RF (0.303) models.

**Figure 2.**
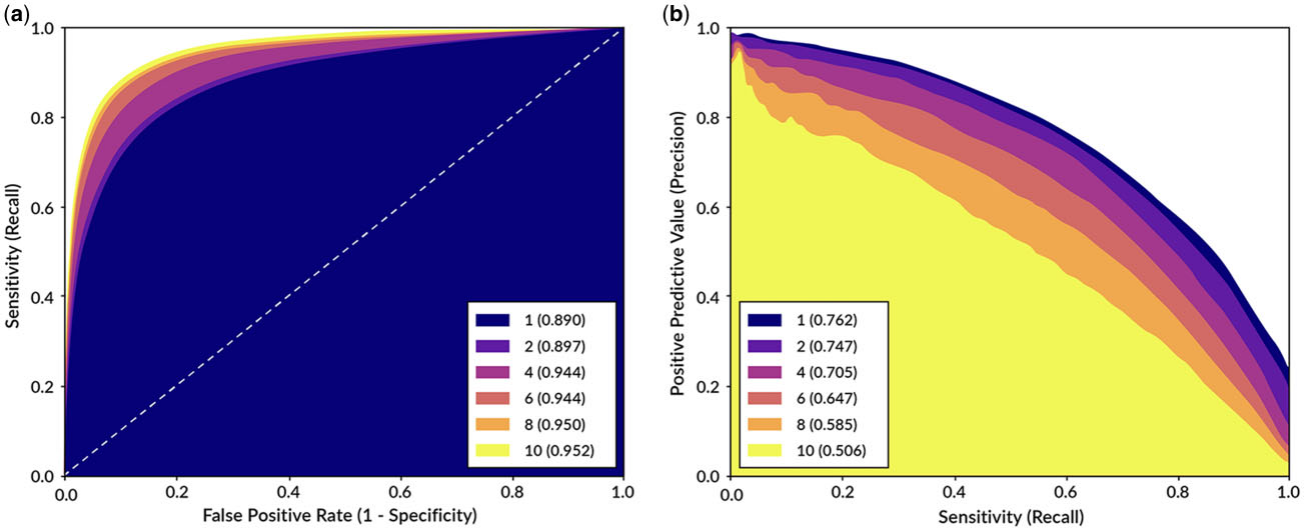
Model performance for thresholds of ≥1, 2, 4, 6, 8, and 10 SABA puffs for (A) the ROC curve (false positive rate vs. sensitivity) and (B) the precision-recall curve. The precision-recall curve is closely associated with the AP metric; values in the legend refer to average precision for each puff threshold.

When examining ROC AUC, the TCN predicted days with ≥1, 2, 4, 6, 8, and 10 SABA puffs on the test group with a score of: 0.890, 0.897, 0.929, 0.944, 0.950 and 0.952, respectively. For higher puff thresholds, performance was higher but relative performance differences were narrower when comparing the TCN vs. alternative models (0.2%-2.1% higher) with this metric. The consistent range but increasing trend of ROC AUC for rarer events stood in contrast to AP, with a wider range and decreasing scores of 0.762, 0.747, 0.705, 0.647, 0.585, and 0.506 for ≥1, 2, 4, 6, 8, and 10 SABA puffs, respectively (Figure 2). This decrease in AP is in part due to the dependence on CPR, which decreases for rarer events. Here the TCN’s performance was also higher than other models, ranging from a 1.8% to 5.1% increase at different thresholds.

**Table 4.** Confusion matrices for (a) symptom days with ≥1 SABA puff and rounded predictions (≥0.5) and (b) high use days with ≥10 SABA puffs and rounded predictions (≥9.5).

		Predicted				Predicted	
	(a)	0	1		(b)	0	1
Actual	0	156 879	34 607	Actual	0	241 112	937
	1	10 589	45 235		1	3446	1815

### Feature interpretation

**Figure 3.**
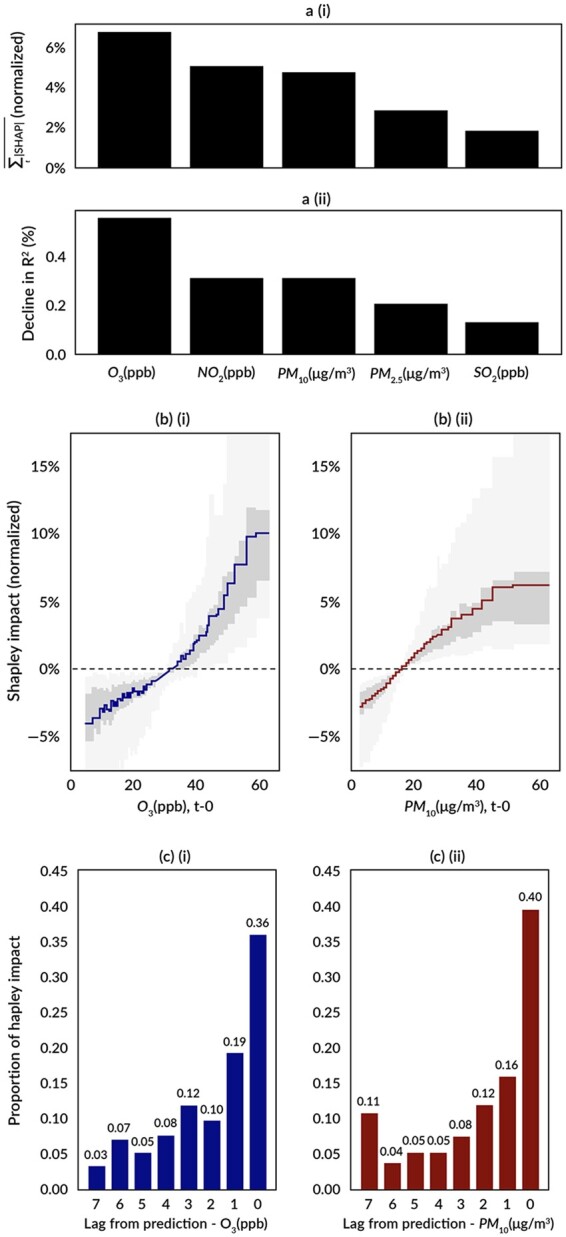
Impact on the model’s predicted SABA response of select criteria pollutants across time lag and concentration. (A) Pollutant feature importance by (i) mean absolute Shapley value (normalized by mean SABA puffs) and (ii) permutation feature importance for variance explained, indicating the % decline in *R*^2^ when that variable is randomly permuted, (B) pollutant concentration distributions from the 1^st^ to 99^th^ percentiles vs. Shapley values normalized by mean SABA puffs, including mean (blue or red line), IQR (dark gray) and 2.5^th^–97.5^th^ percentile range (light gray) at *t*−0 (prediction day) for (i) ozone and (ii) PM_10_, (C) proportion of Shapley impact by lagged day from prediction day (*t*−0) for (i) ozone and (ii) PM10.

Features included in the model, their source, and mean (SD) values are presented in Supplementary Table 1. SABA use in the days prior to prediction was the dominant variable for both permutation-based (with 120% relative decline in *R*^2^) and Shapley-based variable importance (with summed absolute Shapley values across all days equal to 132% of mean SABA use). Environmental factors contributed a small but meaningful impact, particularly at the right tails of their distributions (Figure 3A). Ozone and PM_10_ were chosen for further investigation of their impact across their prediction day distribution and lag days.

By examining Shapley values for the prediction day across the distribution of ozone and PM_10_ concentration levels, we observed a non-linear response in SABA puffs (Figure 3B). Ozone levels above 35 ppb were associated with an increase in SABA use, with positive average Shapley values, along with increasing at a significantly greater rate than levels <35 ppb. While the mean Shapley value toward the right tail of PM_10_ did not exhibit the same increase in rate of change, both pollutants exhibited more extreme outliers in Shapley values (with the 97.5th percentiles representing greater than 15%-20% of mean SABA use) toward the right tail of their concentrations. This suggests that while on average these concentrations are associated with a moderate increase in SABA use, higher pollutant concentrations could contribute to an outlier SABA use event indicating worsening.

The proportion of absolute Shapley values across 0-7-day lags for ozone and PM_10_ also suggests that more than a third of the effect of ozone and PM_10_ on inhaler use is explained by the pollutant concentration on the prediction day, while more than half of the effect is explained by the prediction day plus the day before (Figure 3C). Prior to that, the effect has a noticeable drop off and tends to roughly decrease in magnitude, but does not obviously approach 0, for the preceding 2-7 days.

## Discussion

### Model performance

Model performance results demonstrated that ML models can predict SABA use for a participant-day with accuracy, which is important to understand in both an isolated and relative context.

With a threshold of ≥1 puff/day, the model achieved an AP of 0.762. Given the CPR of 22.6%, this corresponds to more than a threefold increase in the proportion of correct predictions among positive predictions over a random model. This also outperforms the alternative models by a notable margin, which received APs between 0.692 and 0.731, representing relative improvements between 4.3% and 10.1%. Using a higher threshold of ≥10 puffs to correspond to unsafe SABA dosing,^6^^,^^7^ the model reached an AP of 0.506; considering the CPR of 2.1%, this is a 24-fold increase in average precision over a random model. Alternative models scored APs between 0.457 and 0.497, for similar relative improvements between 1.8% and 10.7%.

Further, the TCN meaningfully outperformed alternative models on a relative basis across a range of metrics for predicting participants’ daily SABA puff count. The model also compares favorably in the context of existing efforts to predict asthma and COPD worsening, with ROC AUC scores (the most consistently reported metric) including but not limited to 0.859,^24^ 0.833,^25^ 0.65,^26^ versus 0.890-0.952 for the TCN reported here. However, comparisons remain challenging due to differences across studies in the (1) predicted outcome (eg, SABA vs. exacerbations, ED visits, hospitalizations or readmissions), (2) prediction windows, from 1 day to 1 year, (3) disclosure of CPRs, which have a significant impact on many metrics^27^ and (4) reported performance metrics.

These results suggest that a system involving passive monitoring of SABA use, combined with a predictive model, may provide practical value for both patients and healthcare professionals to monitor and then predict days with worsening symptoms. Furthermore, using more sophisticated modeling approaches, and a rich dataset including participant context such as environmental exposure, demonstrates additional improved predictive performance. Together, these findings suggest promise in leveraging SABA data collected by digital sensors and other contextual data to enable the identification, prediction, and ultimately the prevention of exacerbations, and warrant further validation.

### Feature interpretation

Model development benefited from a large longitudinal health outcome dataset collected passively and objectively from participants in the real-world. The collection of the date, time, and place of SABA use enabled the merging of relevant environmental datasets with the health outcome data, including weather and air pollution data, to begin to evaluate the context in which SABA use occurred.

Using SHAP, we proposed a way to visualize and understand individual variable impact on SABA use. We observed evidence for non-linear responses to two pollutants examined at the prediction day. These responses align with previously published assessments of the impact of ozone on asthma outcomes,^22,^^28–30^ but add nuance as to the non-linearity of the relationship.

Additionally, we observed evidence for the partial effect of lagged pollutant exposures over 7 days on SABA use. These findings are consistent with previous controlled chamber studies,^31^ where an effect was demonstrated in the 3-5 days before the index day. Epidemiological studies have also demonstrated lagged effects of ozone^22^^,^^28^ and PM_10_.^32–35^

### Performance metrics and interpretability methods

We emphasized AP over ROC AUC for two primary reasons. First, AP and the closely related precision-recall curve have been demonstrated to provide a more informative understanding of a classifier’s performance for rare events and imbalanced class distributions.^27^ AP scores exhibited a much wider dispersion across models and puff thresholds and tended to decrease with the rarer events. This contrasts with ROC AUC scores, which tended to remain the same or increase for more frequent events. Second, precision a.k.a. positive predictive value (PPV) can be tied to real-world costs more concretely than specificity due to their respective denominators. Every predicted positive (the denominator of PPV) may incur a cost—for example, an escalation pathway involving a healthcare professional that incurs staff and facility cost—but not every condition negative (the denominator of specificity) may incur a cost.

Combining a TCN with an interpretability method, such as SHAP values, can yield novel quantitative understanding of how different physical and environmental factors influence the response both over time and across the distribution of a variable. Having a granular understanding that can still be interpreted by healthcare practitioners is critical to facilitating long-term adoption of more complex models for healthcare risk assessment and intervention.

### Limitations

While this model development and evaluation is an important step in understanding the role of digital data in predicting respiratory decline, there were several limitations. We lacked rich demographic and other health information on each participant, which limited our interpretations of the model’s predictive ability within different subgroups, some of which are disproportionately affected by respiratory disease. It is also worth noting that the population was largely self-enrolled via social media campaigns and clinics, and there was no standard enrollment process or clinical integration across participants. Inclusion criteria were also broad and included a self-reported diagnosis of asthma. Most participants were female (71.0%), which was slightly higher than the national prevalence among adult women (62.6%).^36^ The majority of participants were uncontrolled upon evaluation at baseline (89.7%), which may limit the generalizability of the findings; however, asthma control is a dynamic status and may have changed throughout the data collection period by season and self-management behaviors. The South and Midwest were overrepresented by 17.6% and 14.1%, and the West and Northeast were underrepresented by 13.0% and 23.5% compared to their respective asthma population sizes.^37^ Race and socioeconomic status (SES) were unavailable for most participants. Previous work has shown SES to be a rough marker for environmental exposures,^38^ which could provide further improvement to the model as well as help assess bias in performance. Further, it is possible that not all SABA usage was captured—oftentimes, patients have multiple inhalers (eg, at home, in the car and at work/school) and it is possible that not all inhalers had a sensor. Thus, the results observed may represent a conservative interpretation of the value of SABA in predicting worsening asthma control. During data processing, we also dropped days with more than 30 puffs instead of capping them as they were assumed to be caused by device error, resulting in less data being captured. The use of air pollution data from the closest regulatory air quality monitors might also bias the air pollution exposure. Air pollution exposure modeling techniques could be applied to derive more accurate air pollution levels at locations of interest. One should also note that Shapley values, though a useful tool for determining post-hoc explanations of predictions, are not designed to capture the importance of groups of features and instead treat them separately.

### Future work

Future work should continue to explore the capacity of deep-learning and other ML models to predict acute respiratory events such as exacerbations. Further research will be conducted to compare periods of elevated SABA use against clinically confirmed asthma-related exacerbation events. Development of individualized thresholds of SABA use that indicate a clinically meaningful worsening event will also be important. Collection and analysis of more diverse training and test sets, including participants across race, ethnicity, gender, and socioeconomic strata will be essential for the relevance and accuracy of these models, especially given the bias demonstrated in AI to date.^14^ Additional signals should also be evaluated.

Future work may seek to further validate some of the approaches to visualize and understand individual variable impact on SABA use—in particular, the stability of the Shapley values, their underlying additive assumption, and comparisons to other interpretability methods. These efforts may respond to the increasing demand at the clinical and regulatory level for increased transparency in AI and ML. For example, labeling requirements from the FDA may require an algorithm’s training data, inputs, logic, use cases, and performance results.^39^

### Conclusion

These findings suggest promise in leveraging SABA data, collected by digital sensors, and other contextual data to enable the identification, prediction, and ultimately, prevention of exacerbations. More research remains to be done to ensure the success of these approaches within a real-world implementation.

## Supplementary Material

ooad091_Supplementary_Data

## Data Availability

The medication use data that supported this study are not publicly available because they are considered Protected Health Information (PHI) under the Health Insurance Portability and Accountability Act of 1996 (HIPAA) in the US, and as such are only accessible under specific authorization of access following HIPAA guidelines.
