# Supplementary material for: Prediction of short-acting beta-agonist usage in patients with asthma using temporal-convolutional neural networks
Source: JAMIA Open. 2023 Oct 26;6(4):ooad091. doi: 10.1093/jamiaopen/ooad091 (PMC10602590; doi:10.1093/jamiaopen/ooad091)
Supplement: ooad091_Supplementary_Data [file ooad091_supplementary_data.docx]

# Supplementary Materials

Supplementary Table 1

|  | Mean (SD) | Source |
| --- | --- | --- |
| NO_2_ (ppb) | 10.26 (7.29) | U.S. Environmental Protection Agency (EPA)’s Air Quality System (AQS) |
| SO_2_ (ppb) | 1.26 (1.72) |  |
| O_3_ (ppb) | 28.24 (12.59) |  |
| PM_10_ (µg/m^3^) | 17.75 (14.27) |  |
| PM_2.5_ (µg/m^3^) | 8.59 (6.36) |  |
| Maximum temperature (°C) | 12.78 (11.63) | National Oceanic and Atmospheric Administration (NOAA) Quality Controlled Local Climatological Data Repository |
| Maximum wind speed (m/s) | 3.29 (1.87) |  |
| Precipitation (mm) | 1.13 (4.97) |  |
| Age | 38.73 (17.12) | Propeller Health, self-report |
| SABA | 0.91 (2.47) | Propeller Health, sensor-collection |

Age and SABA puffs will differ from Table 1 since the values in Supplementary Table 1 are calculated at the sample level, rather than the participant level across the entire dataset.

## Environmental assignment information

Each rescue medication use event or heartbeat event was assigned concentrations of the five criteria pollutants. We included criteria pollutants known to be relevant for asthma, including nitrogen dioxide (NO_2_), ozone (O_3_), sulfur dioxide (SO_2_), and particulate matter of ≤2.5 microns (PM_2.5_) and ≤10 microns (PM_10_). Air pollutant data were acquired from the U.S. Environmental Protection Agency (EPA)’s Air Quality System (AQS). Each user location was assigned weather conditions acquired from the National Oceanic and Atmospheric Administration (NOAA) Quality Controlled Local Climatological Data Repository (QCLCD). Data assigned included hourly measures for air temperature, relative humidity, wind speed, atmospheric pressure, and visibility. If a user did not have any location on a given day, the most recent location available within 24 hours was used. If no location was available within 24 hours, residential location was used to assign daily environmental information. The assigned weather and air pollutant data characterized each participant’s environmental exposures within a day (inclusive of multiple locations within a day) which were then averaged for the day.

## Additional data processing details

Each sample input was created using a trailing window over the previous 7 days along with the prediction day, with SABA inhaler puffs for the prediction day zeroed out to avoid input-output pollution. The window of 7 days was chosen to balance the capture of relevant week-long trends in health against model complexity. Inputs with missing values were dropped from the analysis. In addition, days with over 30 puffs were considered erroneous and dropped. All non-binary variables were normalized by the mean and standard deviation of the training set in each cross-validation fold. Categorical month and weekday variables were one-hot encoded.

## Model development information

Models were trained with mean squared error loss and optimized using the Adam optimizer[[18]](#app_bibliography_4810343808761856) on each of the 3 cross-validation groups. Candidate hyperparameters for the model were evaluated for highest R^2^ on the three cross-validation groups during model selection. The optimal hyperparameters were used to train the final model, now trained on all cross-validation groups.

For feature interpretation with Shapley values, a sample of 2,000 days were randomly selected to calculate a background distribution, and 10,000 days were then randomly sampled for analysis.

## Definitions for evaluation metrics

We define positive predictive value (PPV) in this setting to be the proportion of true positives out of all predicted positives. Sensitivity refers to the proportion of true positives out of all condition positives. Specificity refers to the proportion of true negatives out of all condition negatives. The false positive rate (FPR) refers to the proportion of false positives out of all condition negatives. We examined both average precision (AP) and receiving operator characteristic area under the curve (ROC AUC). AP measures the ability of a classifier to distinguish correctly identify the positive class, and the higher the AP, the better the performance of the model at differentiating between positive (i.e., a SABA puff count equal to or greater than a threshold) and negative classes. It is closely related to the area under the precision-recall curve, and is calculated as the weighted mean of PPV achieved at each probability threshold, weighted by the change in sensitivity from the previous probability threshold:

$$AP=\sum_{n} \left( S_{n}-S_{n-1} \right) P_{n}$$

Where $S_{n}$ denotes the sensitivity at the $n^{th}$ probability threshold. The ROC AUC is another measure of the ability of a classifier to distinguish between classes and is used as a summary of the ROC curve, created by plotting the FPR vs. sensitivity for all possible classification thresholds. Like AP, higher ROC AUC implies better performance of the model at distinguishing between the positive and negative classes.

## Model Architectures and Hyperparameters

The TCN’s hyperparameters consisted of linear and leaky ReLU activation on layers, a kernel size of 2 and max dilation rate of 2 with 64 filters for 1D convolution layers, and skip connections as detailed in Supplementary Figure 1. The random forest model was built with 30 estimators and a maximum tree depth of 8, with squared error as the splitting criterion. The selection of estimators and depth was made to balance computation time and model complexity. Linear regression was performed by minimizing squared error with no regularization.

Supplementary Table 2

| **Model** | **Hyperparameter Name** | **Hyperparameter Value** |
| --- | --- | --- |
| TCN | Input window size | 8 |
|  | Initial learning rate | 0.0003 |
|  | Batch size | 256 |
|  | Layer activations | linear, leaky ReLU |
|  | 1D convolution filters | 64 |
|  | 1D convolution size | 2 |
|  | 1D convolution maximum dilation rate | 2 |
|  | 1D convolution padding | causal |
|  | Loss function | mean squared error |
| Random forest | Number of estimators/trees | 30 |
|  | Maximum tree depth | 8 |
|  | Splitting criterion | squared error |
| Linear regression | Regularization | none |
